# New Concept of the Biosynthesis of 4-Alkyl-L-Proline Precursors of Lincomycin, Hormaomycin, and Pyrrolobenzodiazepines: Could a γ-Glutamyltransferase Cleave the C–C Bond?

**DOI:** 10.3389/fmicb.2016.00276

**Published:** 2016-03-07

**Authors:** Petra Jiraskova, Radek Gazak, Zdenek Kamenik, Lucie Steiningerova, Lucie Najmanova, Stanislav Kadlcik, Jitka Novotna, Marek Kuzma, Jiri Janata

**Affiliations:** Institute of Microbiology – Academy of Sciences of the Czech RepublicPrague, Czech Republic

**Keywords:** anticancer drug, antibiotics, natural product biosynthesis, secondary metabolism, 4-propyl-L-proline, lincomycin, pyrrolobenzodiazepine, hormaomycin

## Abstract

Structurally different and functionally diverse natural compounds – antitumour agents pyrrolo[1,4]benzodiazepines, bacterial hormone hormaomycin, and lincosamide antibiotic lincomycin – share a common building unit, 4-alkyl-L-proline derivative (APD). APDs arise from L-tyrosine through a special biosynthetic pathway. Its generally accepted scheme, however, did not comply with current state of knowledge. Based on gene inactivation experiments and *in vitro* functional tests with recombinant enzymes, we designed a new APD biosynthetic scheme for the model of lincomycin biosynthesis. In the new scheme at least one characteristic in each of five final biosynthetic steps has been changed: the order of reactions, assignment of enzymes and/or reaction mechanisms. First, we demonstrate that LmbW methylates a different substrate than previously assumed. Second, we propose a unique reaction mechanism for the next step, in which a putative γ-glutamyltransferase LmbA indirectly cleaves off the oxalyl residue by transient attachment of glutamate to LmbW product. This unprecedented mechanism would represent the first example of the C–C bond cleavage catalyzed by a γ-glutamyltransferase, i.e., an enzyme that appears unsuitable for such activity. Finally, the inactivation experiments show that LmbX is an isomerase indicating that it transforms its substrate into a compound suitable for reduction by LmbY, thereby facilitating its subsequent complete conversion to APD 4-propyl-L-proline. Elucidation of the APD biosynthesis has long time resisted mainly due to the apparent absence of relevant C–C bond cleaving enzymatic activity. Our proposal aims to unblock this situation not only for lincomycin biosynthesis, but generally for all above mentioned groups of bioactive natural products with biotechnological potential.

## Introduction

**FIGURE 1:**
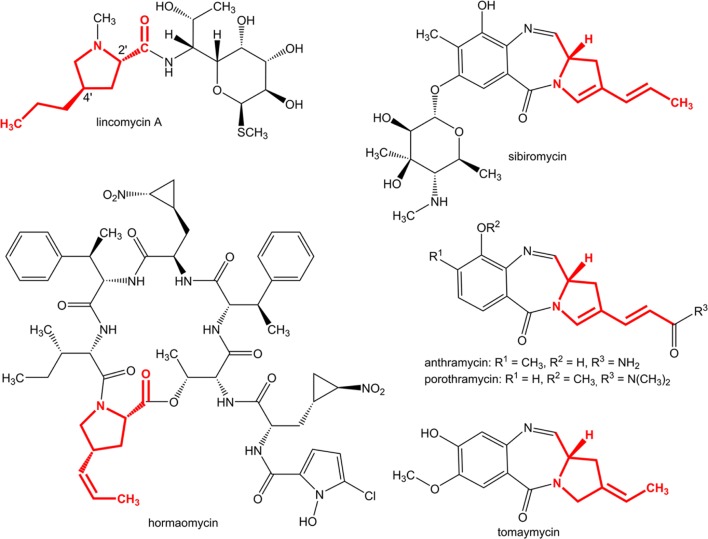
**Natural compounds containing an APD moiety with already known biosynthetic gene clusters (structures originating from APD biosynthesis are in red)**.

4-Alkyl-L-proline derivatives (APDs) are specialized precursors that are common for three groups of structurally different secondary metabolites (**Figure 1**) with various but remarkable biological activities: pyrrolo[1,4]benzodiazepines (PBDs; Leimgruber et al., 1965; Gauze et al., 1969; Kariyone et al., 1971; Tsunakawa et al., 1988) are sequence-selective DNA alkylating agents with significant antitumor properties (for review see Gerratana (2012)), ribosome binding lincomycin (Hoeksema et al., 1964) produced by *Streptomyces lincolnensis* is a clinically used lincosamide antibiotic, and hormaomycin from *Streptomyces griseoflavus* (Rössner et al., 1990) is a microbial hormone that is involved in the quorum-sensing system. Some PBDs and lincosamides incorporate proteinogenic L-proline instead of APD in their structures (e.g., DC-81, neothramycin or tilivalline in PBDs and lincosamide celesticetin), however, these compounds exhibit low biological activity. The length and/or modification of the alkyl chain of the proline moiety affect the efficiency (Magerlein, 1977; Gerratana, 2012). Modification of this parameter, e.g., by mutasynthesis, appears to be an important tool for achieving the production of more efficient derivatives (Ulanova et al., 2010).

**FIGURE 2:**
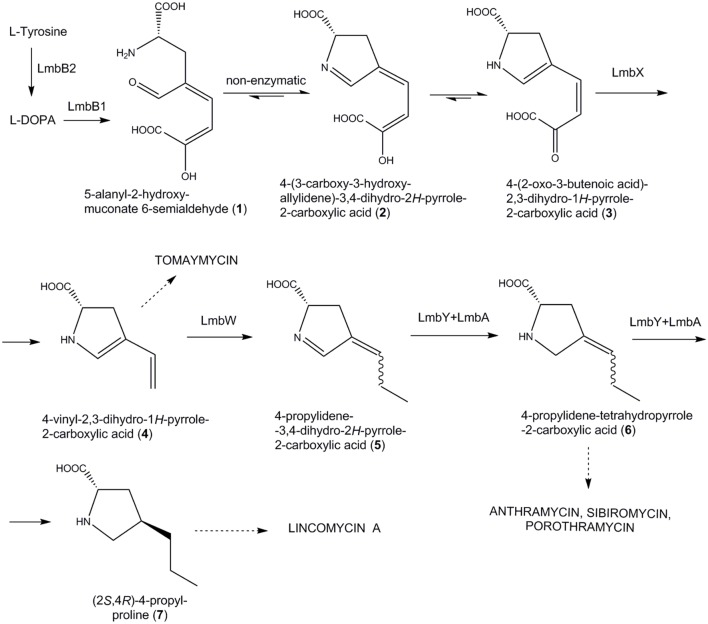
**Previously proposed APD biosynthetic pathway scheme.** According to Li et al. (2009b), adapted for PPL biosynthesis, i.e., sibiromycin biosynthetic proteins were substituted by lincomycin homologs. Dotted arrows indicate branch points of the pathway leading to particular APDs incorporating natural compounds (in capitals).

The APD precursors arise from L-tyrosine through a specialized biosynthetic pathway. Based on feeding experiments with labeled L-tyrosine and L-DOPA in the biosynthesis of lincomycin (Brahme et al., 1984) and some PBDs (Hurley et al., 1979), the general scheme of the APD pathway was proposed (**Figure 2**): L-tyrosine is first converted to L-DOPA, which further undergoes extradiol cleavage to a 5-alanyl-2-hydroxy-muconate 6-semialdehyde (**1**). Following cyclization yields two tautomers, a cyclic imine **2** and a cyclic α-ketoacid **3**. Their equilibrium is strongly shifted in favor of acid **3** (approximately 95:5; Saha et al., 2015). These initial steps were confirmed through a direct biochemical proof (Neusser et al., 1998; Novotna et al., 2004, 2013; Colabroy et al., 2008, 2014; Connor et al., 2011; Saha et al., 2015), but the subsequent steps of this pathway still remain only hypothetical. Generally, it is hypothesized that cleavage of the two carbon residue from the side chain of **3** results in carboxylic acid **4**, which is considered to be the first branch point in the biosynthesis of secondary metabolites containing APD (**Figure 2**; Hurley et al., 1979).

Comparison of relevant biosynthetic gene clusters for APD-incorporating compounds, such as the PBDs anthramycin (Hu et al., 2007), sibiromycin (Li et al., 2009b), tomaymycin (Li et al., 2009a), and porothramycin (Najmanova et al., 2014); lincomycin (Koberska et al., 2008); and hormaomycin (Höfer et al., 2011), reveals the presence of a set of 5–6 homologous genes shared by all mentioned clusters (**Table 1**) and thus assigned to the APD biosynthetic pathway. In contrast, in the clusters for related compounds that incorporate L-proline instead of APD, such as the lincosamide celesticetin (Janata et al., 2015) and the PBD tilivalline (Schneditz et al., 2014), the whole set is missing.

**Table 1:** Homologous proteins involved in the APD biosynthesis shared by producing strains of relevant compounds.

Previously proposed function	Sequence homology to	Lincomycin	Hormaomycin	Anthramycin	Porothramycin	Sibiromycin	Tomaymycin
3-Hydroxylation of L-tyrosine^∗^	L-Tyrosine-3-hydroxylase	LmbB2^∗^	HrmE	ORF13^∗^	POR14	SibU	TomI
Extradiol cleavage of L-DOPA^∗^	L-DOPA-2,3-dioxygenase	LmbB1^∗^	HrmF^∗^	ORF12^∗^	POR13	SibV^∗^	TomH
Cleavage of C–C bond of **3**^∗∗^	**Isomerase**	LmbX	—	ORF15	POR16	SibS	TomK
*C*-Methylation of **4**^∗∗∗^	*C*-Methyltransferase	LmbW	HrmC	ORF5	POR10	SibZ	—
Activation of F420 cofactor^∗∗^	**γ-Glutamyltransferase**	LmbA	HrmG	ORF6	POR11	SibY	TomL
Reductions of **5** to give **7**^∗∗∗∗^	F420-dependent reductase	LmbY	HrmD	ORF14	POR15	SibT	TomJ


*^∗^Function has been proved previously (Novotna et al., 2004, 2013; Connor et al., 2011; Saha et al., 2015). ^∗∗^Previously proposed function not consistent with sequence homology (in bold); novel functions proposed in this paper. ^∗∗∗^Previously proposed function consistent with sequence homology; novel substrate proposed in this paper. ^∗∗∗∗^Previously proposed function consistent with sequence homology; novel mode of action and novel intermediate proposed in this paper.*

In lincomycin biosynthesis, the specific product of the APD pathway, the 4-propyl-L-proline (PPL) precursor, is condensed (Kadlcik et al., 2013; Janata et al., 2015; Zhao et al., 2015) with the amino octose unit, which is a product of another specialized pathway (Sasaki et al., 2012; Lin et al., 2014). The resulting lincosamide scaffold is further modified to the final product, lincomycin A (lincomycin, unless otherwise specified; **Figure 1**; Kamenik et al., 2016). Correspondingly with PPL incorporation, the full set of six APD putative biosynthetic genes is present in the lincomycin biosynthetic cluster (**Table 1**). Two of these genes encode the L-tyrosine hydroxylating enzyme (LmbB2) and L-DOPA-2,3-dioxygenase (LmbB1), catalyzing the first two steps of the PPL pathway (**Figure 2**). Proteins LmbA, LmbW, LmbX and LmbY, encoded by the remaining four genes, thus presumably catalyze all subsequent PPL biosynthetic steps. Seven out of nine carbons of the initial L-tyrosine molecule were demonstrated in the PPL structure, while two are removed and one comes from *S*-adenosylmethionine (Brahme et al., 1984). Accordingly, LmbX was predicted to cleave off the two-carbon oxalyl residue from α-ketoacid **3** and LmbW to methylate the resulting compound **4** (Li et al., 2009a,b), yielding characterized intermediate **5** (Kuo et al., 1992). While the methylation function of LmbW was recently documented by inactivation of the coding gene (Pang et al., 2015), in the case of LmbX, the predicted C–C cleavage function is contradictory: (1) LmbX belongs, based on sequence homology, to the isomerase protein family and (2) the LmbX homolog is missing in the hormaomycin biosynthesis (Höfer et al., 2011), but the structure of hormaomycin does not correspond with the lack of the C–C cleavage functionality. Lincomycin and hormaomycin both incorporate the APD precursor with the same length of alkyl side chain but with different levels of saturation. Moreover, the proposed function of LmbA (activation of cofactor for F420-dependent reductase LmbY, where the glutamate ligase activity should be expected) does not correspond to its sequence homology to γ-glutamyltransferases (EC 2.3.2.2; Castellano and Merlino, 2012).

The aim of this work was to revise the APD biosynthetic pathway on the lincomycin model based on recent literature data and our results from gene inactivation experiments and functional testing of individual proteins with compound **3** as a substrate.

## Results

### Production of Lincomycin Derivatives and Amino Acid Precursors by *S. lincolnensis* and its Deletion Mutant Strains

#### Overview of Detected Compounds

**FIGURE 3:**
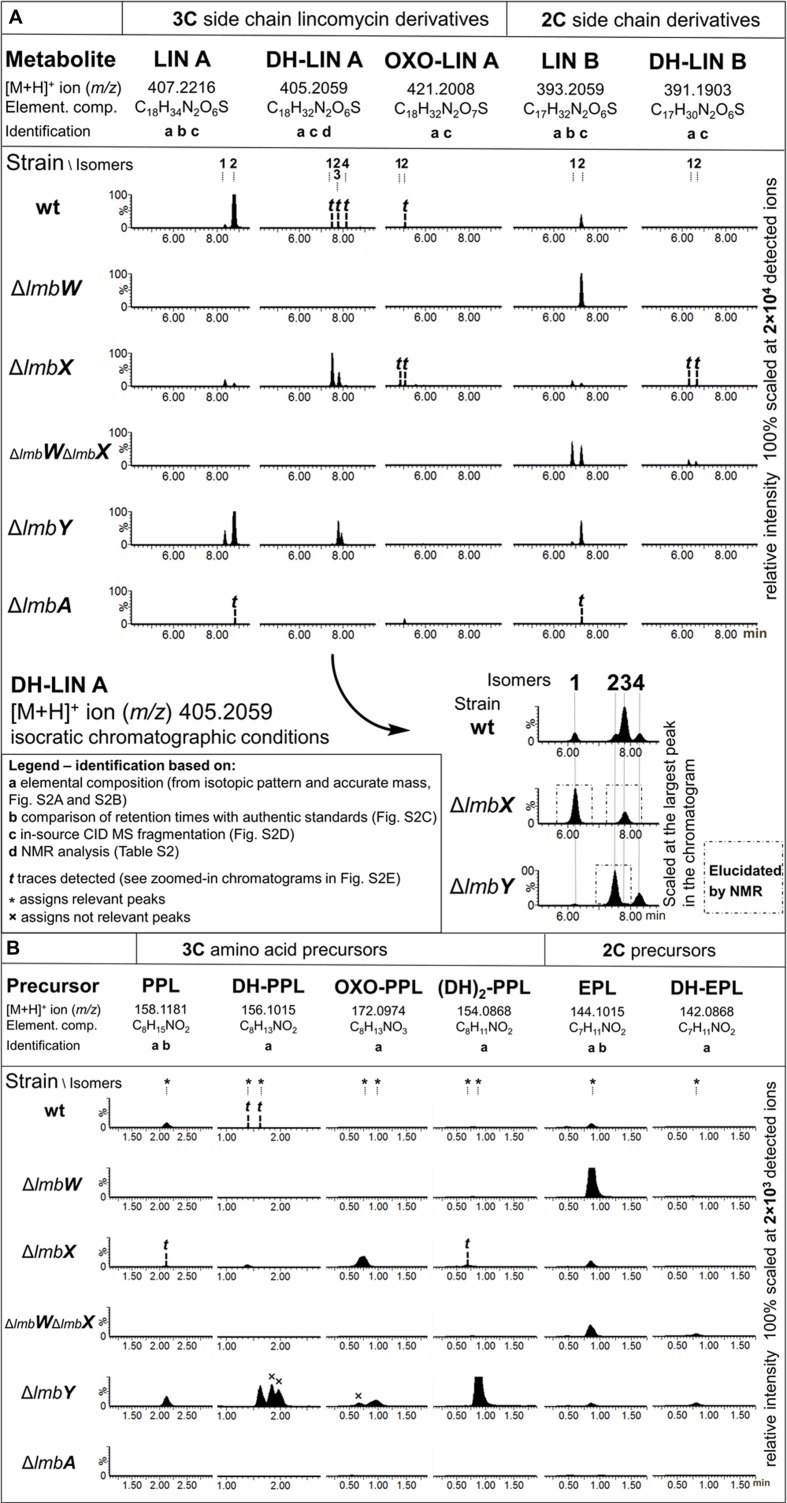
**Production profiles of wt and deletion mutant strains.** **(A)** Lincomycin derivatives and **(B)** Amino acid precursors. LC–MS ion extracted chromatograms; Extraction window: 0.025 Da. Not relevant peaks (assigned with ×) do not represent [M+H]^+^, but a fragment of different species, or their mass error exceeds 5 ppm compared to the theoretical elemental composition (see also **Supplementary Figures S1A–E**).

**FIGURE 4:**
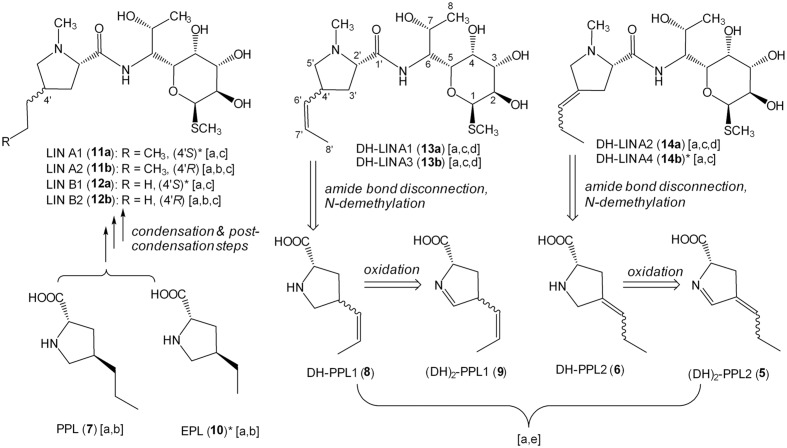
**Structures of identified products and precursors found in culture broths of wt and deletion mutant strains and their biosynthetic relations.** Letters in square brackets indicate methods used for corresponding structure identification: a – elemental composition from isotopic pattern and accurate mass (**Supplementary Figures S1A,B**); b – comparison of retention times with authentic standards (**Supplementary Figure S1C**); c – in-source CID MS fragmentation (**Supplementary Figure S1D**); d – NMR analysis (**Supplementary Table S1**); e – retro-(bio)synthetic approach (indicated by retrosynthetic arrows starting from completely identified products **13a**, **13b**, and **14a**). ^∗^Tentative structure (absolute or relative configuration is proposed based on the knowledge of the major isomer structure).

*Streptomyces lincolnensis* ATCC 25466 (wt strain) and its mutant strains with a relevant gene(s) deleted were cultured and analyzed for the production of lincomycin, its derivatives and corresponding amino acid precursors (summarized in **Figure 3**). Relevant structures are shown in **Figure 4**. In addition to lincomycin A (LIN A) bearing a three-carbon side chain (3C; n-propyl) at the C-4′ position of the amino acid moiety and the side product lincomycin B (LIN B), bearing a two-carbon side chain (2C; ethyl) at the same position (**Figure 4**), we found several previously undescribed lincomycin derivatives with a modification in their amino acid moiety: DH-LIN A, DH-LIN B, and OXO-LIN A. Moreover, we searched for compounds corresponding to the amino acid precursors of LIN A and LIN B, PPL, and 4-ethyl-L-proline (EPL), respectively (**Figure 4**); for precursors of the newly identified lincomycin derivatives, DH-PPL, DH-EPL, and OXO-PPL; and finally for the previously identified intermediate (DH)_2_-PPL (Kuo et al., 1992). All of these compounds are defined by their elemental composition (**Supplementary Figures S1A,B**), and their abbreviations indicate the difference compared to LIN A, LIN B, PPL, or EPL: DH and (DH)_2_ represent the presence of one or two double bonds, respectively, and OXO indicates the presence of one additional O and two less H atoms. Further identification (comparison with authentic standards, MS fragmentation, and NMR) is shown in **Figure 3** and evidenced in **Supplementary Figures S1C,D** and **Supplementary Table S1**. The production profiles provide useful clues for probing the PPL biosynthetic machinery. Therefore, they are described in further detail below, and their magnified versions are depicted in **Supplementary Figure S1E**.

The nomenclature of isomers was based on their retention order. For the LIN A example, a stereoisomer with the 2′*S*,4′*R* configuration (CAS number 154-21-2) was detected as the later eluting predominant product of wt strain and termed LIN A2 (**11b**). However, another earlier eluted minor stereoisomer of LIN A, termed LIN A1 (**11a**), presumably having the 2′*S*,4′*S* configuration, was detected (**Figures 3A** and **4**).

#### Production Profile of ΔlmbW Strain

The *lmbW* gene inactivation resulted in abolition of LIN A production, and no other lincomycin derivatives with the 3C side chain were detected. Nevertheless, the production of LIN B was unaffected or even increased, and accordingly, only significant accumulation of the relevant EPL precursor (**10**) was detected.

#### Production Profile of ΔlmbX Strain

As the main product of the Δ*lmbX* strain, we detected two DH-LIN A isomers: DH-LIN A1 (**13a**) and DH-LIN A3 (**13b**). Their structures were confirmed by NMR analysis (**Supplementary Table S1**), revealing that both isomers contain a double bond, located in the middle of the 3C side chain in a *Z-*configuration (**Figure 4**). The two isomers differ in absolute configuration at either C-2′ or C-4′; C-4′ being more probable because it corresponds with utilization of L-tyrosine as a starting compound. The production of other lincomycin derivatives was low, and all the compounds were produced in two isomers with comparable ratios or favoring the earlier eluting isomers. Additionally, these isomers likely differ in terms of the absolute configuration at C-4′. Furthermore, we detected precursors PPL (**7**), EPL (**10**), DH-PPL1 (**8**), and OXO-PPL, corresponding to the lincomycin derivatives produced by this strain, and we also detected a low amount of (DH)_2_-PPL1 (**9**; **Figure 4**). The structures of relevant unsaturated precursors **8** and **9** were derived based on retro-(bio)synthetic analysis of determined products **13a** and **13b** (**Figure 4**). When complemented with plasmid-bearing *lmbX* gene under control of a constitutive promotor, the Δ*lmbX* strain restored the production profile of the wt strain (data not shown).

#### Production Profile of ΔlmbWΔlmbX Strain

Similar to single *lmbW* gene inactivation, no lincomycin derivatives with the 3C side chain were detected. Only LIN B and DH-LIN B were produced, both in two isomers, resembling the production profile of the Δ*lmbX* strain. The corresponding precursors, EPL and DH-EPL, were also detected.

#### Production Profile of ΔlmbY Strain

The production of LIN A and LIN B was not abolished. However, the concentration of the main product LIN A2 decreased considerably compared to the wt strain (wt: 35 ± 15 μg/ml; Δ*lmbY*: 10 ± 5 μg/ml), as quantified based on UV detection. Furthermore, we detected two isomers of DH-LIN A, DH-LIN A2 (**14a**) and DH-LIN A4 (**14b**), with different retention times compared to DH-LIN A1 (**13a**) and DH-LIN A3 (**13b**) produced by the Δ*lmbX* strain (see separation of all DH-LIN isomers under isocratic conditions in **Figure 3A**). The structure of DH-LIN A2 (**14a**) was elucidated by NMR (**Supplementary Table S1**), revealing that the double bond is exocyclic, attached to the C-4′ carbon (**Figure 4**). However, unambiguous determination of its relative configuration on the basis of NMR data was not possible. Apart from the precursors of the identified lincomycin derivatives, i.e., PPL (**7**), DH-PPL2 (**6**), and EPL (**10**), we observed a high accumulation of (DH)_2_-PPL2 (**5**; **Figure 4**) without any relevant lincomycin-derivative product. Structures **5** and **6** were derived from product **14a** analogously to intermediates **8** and **9** (**Figure 4**).

#### Production Profile of ΔlmbA Strain

Only trace amounts of LIN A and LIN B were detected. OXO-LIN A was found as the main product, but in a low amount. All identified products were present in single-isomer form: LIN A2, LIN B2, and OXO-LIN A2. Additionally, we did not detect any lincomycin precursors. When complemented with plasmid-bearing *lmbA* gene under control of a constitutive promotor, the Δ*lmbA* strain restored the production profile of the wt strain (data not shown).

### Functional Tests with Recombinant LmbX and LmbW Proteins

#### LmbX did not Cleave Oxalyl Residue of Intermediate **3**

Protein LmbX was proposed to catalyze the cleavage of the C–C bond, resulting in the release of oxalyl residue from **3** (Li et al., 2009b). We tested soluble LmbX protein under various reaction conditions with **3** as a substrate (see Experimental procedures). However, we did not detect cleavage of the oxalyl residue or any other turnover of **3** caused by LmbX.

#### LmbW Methylated Intermediate **3**

**FIGURE 5:**
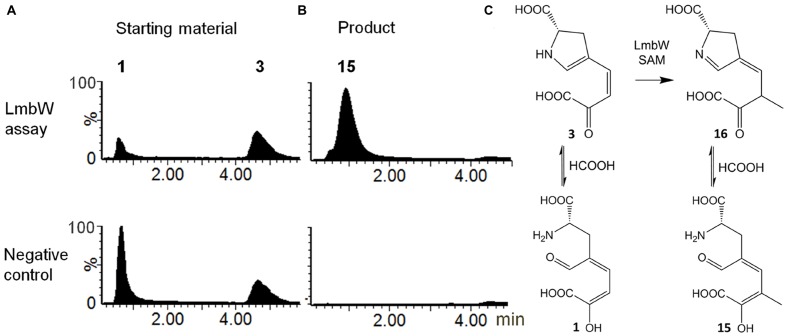
**Conversion of 3 by recombinant LmbW.** **(A)** Starting material: LC–MS ion extracted chromatogram at *m/z* 210.0402 – [M-H]^-^ of **3** and [M-H-H_2_O]^-^ of **1**. **(B)** Product: LC–MS ion extracted chromatogram at *m/z* 242.0665 – [M-H]^-^ of **15**. **(C)** Methylation catalyzed by LmbW including equilibrium with hydrolyzed forms of starting material and product. UV and MS spectra of substrate(s) and the product are shown in **Supplementary Figure S2**.

Recombinant protein LmbW was tested with **3** as a substrate. However, under acidic chromatographic conditions, the starting material **3** was detected as a mixture of **3** and apparently its early eluting acyclic form **1**, likely resulting from hydrolysis of the enamine group of **3** (**Figure 5A**; evidenced by MS and UV spectra in **Supplementary Figure S2**). Indeed, this sensitivity of **3** to acidic hydrolysis has been previously demonstrated (Colabroy et al., 2008; Saha et al., 2015). In the reaction with LmbW and *S*-adenosylmethionine, we identified a product (**Figure 5B** and **Supplementary Figure S2**) with the elemental composition C_10_H_13_NO_6_. The data are consistent with the product being **15**, i.e., methylated **1**. The assumption that the product has its enamine group hydrolyzed is supported by the similar polarities of **15** and **1** and the considerably lower polarity of **15** compared to **3**. Additionally, both compounds **1** and **15** presented similar MS spectra patterns with a significant (**15**) or even predominant (**1**) fragment of loss of water, corresponding to the loss of hydroxyl present in both enol tautomers **1** and **15** (**Supplementary Figure S2**). We assume that **1** and **15** were detected as a consequence of the acidic conditions used during the chromatographic analysis, and it is actually **3**, which is methylated to afford **16** in an *in vivo* system (**Figure 5C**).

### A New Concept of APD Biosynthetic Pathway

#### Contradictory Steps of the Previously Proposed Biosynthetic Pathway

In addition to those mentioned in the introduction, our experimental results revealed several further contradictions in terms of the currently accepted theoretical scheme of APD biosynthesis (**Figure 2**):

**LmbX** does not cleave an oxalyl residue from intermediate **3**. Accordingly, deletion of *lmbX* gene resulted in products with incorporated 3C unsaturated APD moiety, similar or even identical as in hormaomycin (its producer lacks LmbX homolog).

**LmbW** unexpectedly methylates intermediate **3**.

**LmbA** does not activate F420 cofactor of LmbY reductase (Peschke et al., 1995), because the production profiles of the Δ*lmbY* and Δ*lmbA* strains are completely different. Moreover, deletion of the *lmbA* gene resulted in the most efficient block in PPL biosynthesis among all putative APD biosynthetic genes tested, indicating that LmbA catalyzes a key step in the PPL pathway.

**FIGURE 6:**
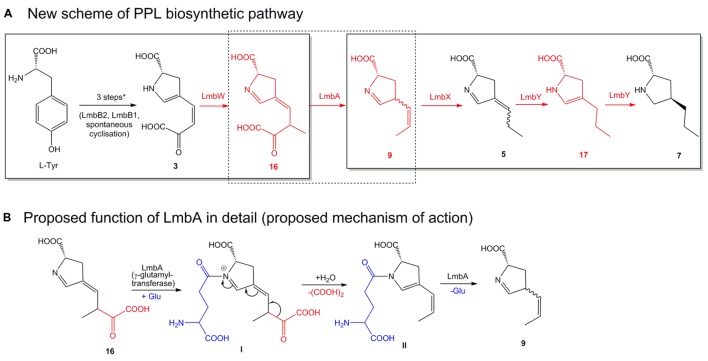
**Newly proposed scheme of the PPL biosynthetic pathway.** The overall PPL biosynthetic scheme **(A)** was elucidated discontinuously in two parts marked by solid-line frames. New structures and newly proposed protein functions are in red. ^∗^ Initial three steps see **Figure 2**. Biosynthesis of EPL differs only slightly, methylation step catalyzed by LmbW is missing, compound **3** is directly cleaved by LmbA to analog of **9** containing ethyliden side chain instead of propyliden, further steps are similar as in PPL biosynthesis, but the compounds **5**, **17**, and **7** are substituted by analogs with the 2C side chain. **(B)** The deduced mechanism of action of γ-glutamyltransferase LmbA (corresponds to dotted frame in **A**). Roman numerals indicate reaction intermediates (see Discussion III).

These contradictions provided us with convincing evidence that the originally proposed biosynthetic scheme (**Figure 2**) required a revision. Moreover, it encouraged us to design a new scheme for PPL biosynthesis (**Figure 6A**) that assigns functions to all the remaining proteins whose functions have not yet been confirmed. Specifically, we have assigned new functions to LmbA and LmbX and a new substrate to LmbW, while the role of LmbY remained basically the same as was originally proposed, i.e., reduction of double bonds of **5**. The newly proposed biosynthetic scheme is fully consistent with published data and with our results, and its individual steps are discussed in further detail below.

## Discussion

### I. Methylation Catalyzed by LmbW (Conversion of 3 to 16)

The *in vitro* methylation of **3** by recombinant LmbW led to its conversion into a product **16** (observed due to an acidic hydrolysis of enamine in its acyclic form **15**). Theoretically, the substrate specificity of LmbW can be relaxed, and therefore, we cannot exclude the previously proposed intermediate **4** (Hurley et al., 1979) from being the main natural substrate. However, we consider this alternative unlikely for the following reasons: (1) The methylation of both **3** and **4** by LmbW would require the existence of two different mechanisms of action – the methylation of enolate in **3** versus the methylation of the conjugated system of double bonds in **4**. (2) The methylation of **4** cannot explain the presence of the two different forms of (DH)_2_-PPL (**5** and **9**), as documented by us in mutant strains Δ*lmbX* and Δ*lmbY*, because it should lead to only a single form **5** (**Figure 2**). (3) Two proteins with significant sequence homology to LmbW, MrsA (26% identity with coverage of 74% according to blastp; Braun et al., 2010) and MppJ (26% identity, 78% coverage; Huang et al., 2009), were demonstrated to be *C*-methyltransferases of compounds sharing structural features with **3** (methylation occurs at the α-position to the carbonyl group of pyruvate residue). (4) Intermediate **4** has never been detected.

The production of lincomycin derivatives not only complies with the methyltransferase function of LmbW but they also reveal that LmbW is dispensable for the PPL/lincomycin biosynthetic machinery. First, *lmbW* deletion abolished the production of all 3C lincomycin derivatives, but not 2C derivatives (LIN B in Δ*lmbW* and Δ*lmbW*Δ*lmbX* strains and DH-LIN B in the latter one, **Figure 3A**), which means that all enzymes catalyzing the following steps in PPL biosynthesis, i.e., LmbA, LmbX, and LmbY, as well as all condensation/post-condensation proteins (Kadlcik et al., 2013; Janata et al., 2015; Zhao et al., 2015; Kamenik et al., 2016), exhibit a relaxed substrate specificity and accept to a certain extent substrates with both two- and three-carbon side chains. Second, a natural system missing the LmbW homolog (biosynthesis of the PBD tomaymycin) analogously incorporates a 2C APD precursor into the final product (Li et al., 2009a). Third, this *C*-methylation step can be omitted even in strains with functional LmbW (for detailed explanation see legend of **Figure 6**). Thus, 2C lincomycin derivatives, particularly LIN B, were detected along with the 3C ones in all the tested strains.

It is challenging to clarify the fate of the LmbW product **16** when considering expected functions of the remaining LmbX, LmbY, and LmbA proteins. However, the results of inactivation experiments and recently published data provide a realistic basis for the elucidation of the final steps of PPL biosynthesis catalyzed by LmbX and LmbY (Discussion part II). Only having explained this part of PPL biosynthesis, we are able to deal with the conversion of **16** (Discussion part III).

### II. Reduction of Double Bonds: The Role of LmbX and LmbY (Conversion of 9 to 7)

**FIGURE 7:**
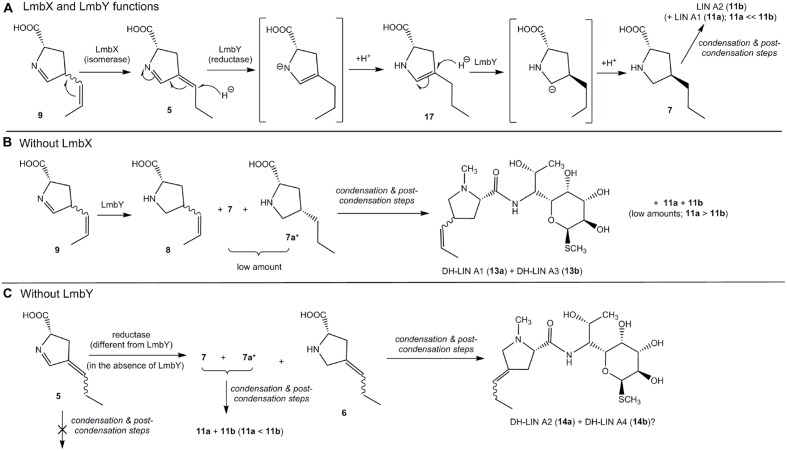
**Functions of LmbX and LmbY.** **(A)** Proposed mechanism of LmbX and LmbY functions (wt strain); square brackets indicate unstable intermediates of reactions. **(B)** Proposed pathway leading to DH-LIN A1 (**13a**) and DH-LIN A3 (**13b**) in Δ*lmbX*; **(C)** Proposed pathway leading to DH-LIN A2 (**14a**) in Δ*lmbY*. ? – structure of DH-LIN A4 (**14b**) is unknown; however, it could be the second stereoisomer of **14a** (at double bond of amino acid moiety); ^∗^ – presence of this compound is expected only, because **7** is not distinguished in LC–MS chromatograms.

Protein LmbY exhibits significant homology to F420-dependent oxidoreductases and is supposed to catalyze two reduction steps, converting intermediate **5** to PPL (**7**; Kuo et al., 1992; Peschke et al., 1995). Indeed, the major accumulated intermediate in ΔlmbY mutant strain was compound with two double bonds [(DH)_2_-PPL2 – presumably intermediate **5**; **Figures 3B** and **4**]. However, except this expected intermediate, two other minor intermediates [DH-PPL2 (**6**) and PPL (**7**)] were also found in the cultivation broth of this mutant strain. Their formation can be explained by either partial or full reduction of **5** by an alternative, less selective (and less efficient) reductase, which is likely encoded outside the lincomycin biosynthetic cluster (**Figure 7C**). The presence of unsaturated intermediates (DH)_2_-PPL1 (**9**) and DH-PPL1 (**8**) in the ΔlmbX mutant strain, indicating seeming LmbX participation in the reduction of double bond(s), appears to be unexpected as well, because LmbX exhibits no sequence homology to oxidoreductases. LmbX is homologous to PhzF protein (27% identity with coverage of 94% according to blastp), which has been proven to be an isomerase (Blankenfeldt et al., 2004; Parsons et al., 2004). As apparent from the production profile of the ΔlmbX mutant strain, the reduction of the endocyclic double bond present in **9** proceeds quite readily, and only trace amounts of this intermediate were detected. However, the complete reduction to PPL (**7**) is inefficient and results in the production of unsaturated lincomycin derivatives (**13a** and **13b**) from corresponding precursor **8** (**Figure 3**). It indicates that LmbY is able to effectively reduce exocyclic double bond in **5**, but not the side chain double bond in **9**. An isomerase activity could solve this situation by converting **9** to its isomer **5**, which represents a more suitable substrate of LmbY. We hypothesize this role for the putative isomerase LmbX.

LmbY presumably utilizes F420 cofactor, which is able to transfer the hydride ion (Schauer et al., 1986). Hydride reduction requires generally the presence of a polarized double bond, and thus, reduction of the polarized endocyclic double bond (imine) should occur. However, intermediate **5** contains the second double bond in conjugation with imine. This mutual position of double bonds enables the so-called conjugate addition of hydride ion to the more distant exocyclic double bond, affording intermediate **17**, which is further reduced to PPL (**7**) by reduction of polar endocyclic double bond (**Figure 7A**). In contrast, intermediate **9** does not allow this conjugate addition and its reduction proceeds predominantly at endocyclic double bond (imine), affording **8** (**Figure 7B**). The side chain double bond in **8** is further reduced very unwillingly (it is non-polar) and **8** will predominate compared to **7** in the Δ*lmbX* strain. Therefore, the strains lacking LmbX or its homolog (Δ*lmbX* strain and hormaomycin producer) predominantly synthesize products with precursor **8** incorporated in their structures – DH-LIN A1 (**13a**), DH-LIN A3 (**13b**) and hormaomycin.

The rate of incorporation of individual unsaturated precursors to final biosynthetic products (lincomycin and its derivatives) indicates another important finding, the substrate specificity of the condensation and post-condensation lincomycin biosynthetic system. Apparently, this system is able to incorporate not only saturated precursors [PPL, EPL; in accordance with Kadlcik et al. (2013)] but also mono-unsaturated ones containing double bond located in the side chain (**6** and **8**). On the other hand, the lincomycin derivatives incorporating APD precursor containing two double bonds have not been observed at all even though at least **5** was accumulated by Δ*lmbY* mutant strain. However, it is impossible to decide, whether the endocyclic double bond itself or its combination with the side chain double bond prevents incorporation.

### III. Cleavage of Oxalate Catalyzed by LmbA (Conversion of 16 to 9)

Compounds **16** (**Figure 6A**, likely the product of the methylation of **3** catalyzed by LmbW) and **9** (probable substrate of LmbX) constitute two distinct intermediates in the biosynthetic pathway of APDs with relatively high structural similarity. Theoretically, a single reaction would be sufficient to connect both intermediates to a substrate-product pair and thus to complete the entire biosynthetic pathway (**Figure 6A**). This reaction represents the already predicted cleavage of the oxalyl residue. However, this cleavage will not occur from previously proposed intermediate **3** but rather from **16** (for 3C APD).

The only remaining putative PPL biosynthetic protein with an unassigned function is LmbA (assuming no dual function of any of the preceding or following proteins LmbB2, B1, W, X, and Y). Bioinformatics analysis of LmbA does not indicate any apparent relevant C–C cleavage activity for a similar transformation because it belongs to the family of γ-glutamyltransferases (Castellano and Merlino, 2012). However, the sequence homology corresponds neither to the previously proposed function of the LmbA protein, i.e., attachment of the glutamyl residue in the biosynthesis of F420 cofactor, which is required for F420-dependent reductase LmbY (Peschke et al., 1995; Li et al., 2009b). This reaction is generally catalyzed by coenzyme F420:L-glutamate ligase (Li et al., 2003), which does not exhibit any homology to γ-glutamyltransferases. The participation of the LmbY and LmbA proteins in the same biosynthetic step is also unambiguously disproved by the distinct production profiles of the Δ*lmbY* and Δ*lmbA* strains (**Figure 3**). Considering all these facts, we should contemplate the possibility that LmbA could cleave **16** through some yet unknown mechanism.

Here, we propose a hypothetical mechanism that can solve this rebus using only the expected γ-glutamyltransferase activity of LmbA (**Figure 6B**). According to our hypothesis, the cleavage of **16** will be initiated by glutamate transfer to the imine nitrogen of **16**, leading to a positively charged nitrogen atom. This should lead to destabilization of intermediate **I**, resulting in an extensive shift of double bonds across a large part of the molecule, ultimately leading to the cleavage of oxalate. Subsequently, the glutamyl residue from intermediate **II** is removed, presumably by LmbA, to afford **9**.

OXO-LIN A, an oxygen-containing derivative of lincomycin, which is produced as the main product by the Δ*lmbA* strain as well as in trace amounts by several other strains (**Figure 3A**), is likely formed by the spontaneous decarboxylation of intermediate **3** (we observed significant decarboxylation of **3** in the presence of metal ions – data not shown), followed by reduction of double bonds (for details of the proposed pathway see **Supplementary Figure S3**). The low or only trace amount of OXO-LIN A production also confirms that this mechanism represents only a shunt pathway, which is not competitive with the natural biosynthesis.

## Materials and Methods

### Bacterial Strains and Chemicals

The type strain *S. lincolnensis* ATCC 25466 producing lincomycin was used for the preparation of all mutant strains. Routine DNA manipulations were performed in *Escherichia coli* JM109 (Promega). The heterologous expression of *S. lincolnensis* genes was performed in *E. coli* BL21(DE3; Novagen). *E. coli* ET12567, BW25113, GM2929, and DH5α strains were used for gene inactivation.

Lincomycin A (containing lincomycin B as an impurity) was purchased from Sigma-Aldrich (Germany), and the standards of PPL, EPL and L-4-(2-oxo-3-butenoic-acid)-4,5-dihydropyrrole-2-carboxylic acid (**3**) were prepared as described in the literature (Novotna et al., 2004; Kamenik et al., 2009). Acetonitrile and methanol (both LC–MS grade) were obtained from Biosolve BV (Netherlands), formic acid (98–100%; Merck KGaA, Germany), and ammonium hydroxide (28–30% A. C. S. reagent; Sigma-Aldrich, Germany).

### Gene Inactivation

Selected genes encoding proteins involved in the PPL pathway were inactivated by the introduction of apramycin resistance cassette pIJ773 using the REDIRECT technology kit (Plant Bioscience Limited, Norwich Research Park, UK) for PCR targeting (Gust et al., 2004). The inactivation and checking primers are listed in **Supplementary Table S2**. To construct Δ*lmbA*, Δ*lmbW*, Δ*lmbX* and Δ*lmbY* mutants, the inactivation cassette was replaced by an 81-nt-long in-frame scar. Double mutant Δ*lmbW*Δ*lmbX* was prepared by the introduction of a hygromycin resistance cassette from pIJ798 instead of *lmbW* and the apramycin resistance cassette from pIJ773 instead of *lmbX*.

### Cultivation of *S. lincolnensis* Strains

The seed culture of *S. lincolnensis* strains was prepared by inoculation from MS plates into 40 ml of YEME medium (Kieser et al., 2000) without sucrose and incubated in 500 ml flat-bottom boiling flasks at 28°C. Two milliliters of 24 h seed culture of *S. lincolnensis* were transferred into 40 ml of AVM medium (Najmanova et al., 2014) and incubated in 500 ml flat-bottom boiling flasks at 28°C for 120 h. The cells were centrifuged at 5000 *g* at 4°C for 15 min, and the supernatant was used for solid-phase extraction.

### Complementation of *S. lincolnensis* ΔlmbA and ΔlmbX

The *lmbA* and *lmbX* genes were PCR amplified from the LK6 cosmid (Koberska et al., 2008) using the primer pairs fA10257N/rA10257N and ExXf/ExXr, respectively (**Supplementary Table S2**). The appropriate amplified sequence was inserted via the NdeI and XhoI restriction sites into an integrative vector pIJ10257 (Hong et al., 2005). The resulting construct was introduced into non-methylating strain *E. coli* ET12567/pUZ8002 and then introduced into the genome of *S. lincolnensis* Δ*lmbA* (for the construct containing *lmbA*) or Δ*lmbX* (for the construct containing *lmbX*) via intergeneric conjugal transfer. Exconjugants were selected with hygromycin (0.1 g/l).

### Heterologous Production of LmbX and LmbW Proteins in *E. coli*

Genes *lmbX* and *lmbW* were PCR amplified from LK6 cosmid using primer pairs ExX and ExW (**Supplementary Table S2**). The products were inserted into pET28b(+; *lmbX*) or pET42b(+; *lmbW*) vector (Novagen). The resulting constructs were used to produce soluble proteins in *E. coli* BL21(DE3; Novagen); LmbX required co-expression with GroES and GroEL chaperonins. The proteins were produced and purified as was described for LmbC (Kadlcik et al., 2013) with the following modifications. The post-induction production of LmbX was conducted at 17°C for 20 h; LmbX required 20% glycerol in all buffers. The protein was dialyzed overnight against TS-8 buffer (Kadlcik et al., 2013) containing 20% glycerol and immediately used. The post-induction production of LmbW was performed at 28°C for 4 h. The protein was dialyzed against 40 mM glycine buffer pH 9.5 and immediately used.

### *In Vitro* and *In Vivo* Assays with LmbX

The enzymatic activity of LmbX was tested with **3** as a substrate in the following reaction mixtures: LmbX (1 mg/ml), **3** (10 mM) and 20% glycerol in a buffer. Several buffers were tested: 20 mM Tris-HCl pH 7, 8, and 9 with and without 100 mM NaCl and 100 mM phosphate buffer pH 7, 8, and 9 with and without 100 mM NaCl. Moreover, the addition of various metal ions was tested: Ca^2+^, Cu^2+^, Fe^3+^, Mg^2+^, Mn^2+^, and Zn^2+^ (10 mM). The reactions with a total volume of 100 μl were incubated at 28°C for 1 h, terminated with 1 μl of formic acid and subsequently analyzed using LC–MS. An *in vivo* experiment was also conducted. Compound **3** (5 mM) was added to bacterial culture BL21(DE3; 6 ml) expressing the *lmbX* gene under the same conditions that were used for the heterologous production of LmbX. The culture medium and supernatant from sonicated cells were analyzed using LC–MS.

### *In Vitro* Assays with LmbW

The enzymatic activity of LmbW with **3** as a substrate was tested in reaction mixtures containing LmbW (1 mg/ml), **3** (4 mM), and *S*-adenosylmethionine (4 mM) in glycine buffer (40 mM, pH 9.5). The reactions with a total volume of 100 μl were incubated at 28°C for 1 h, terminated with 1 μl of formic acid and analyzed using LC–MS.

### Extraction of Amino Acid Precursors

Oasis MCX 3cc 60 mg cartridge (mixed-mode cation exchange sorbent, Waters, USA) was conditioned with 3 ml methanol, equilibrated with 3 ml 2% formic acid and then 3 ml cultivation broth (pH adjusted to 2.3 with formic acid) was loaded. Subsequently, the cartridge was washed with 3 ml water and absorbed substances were eluted with 1.5 ml methanol:water:ammonium hydroxide solution (50:48.5:1.5 v/v/v). The eluent was evaporated to dryness, reconstituted in 300 μl methanol:water (1:1 v/v) and centrifuged at 13000 rpm for 5 min. Two μl of the extract were injected into LC–MS.

### Extraction of Lincomycin Derivatives

Oasis HLB 3cc 60 mg cartridge (hydrophilic-lipophilic balanced sorbent, Waters, USA) was conditioned with 3 ml methanol, equilibrated with 3 ml water and then 3 ml cultivation broth (pH adjusted to 9.0 with ammonium hydroxide) was loaded. Subsequently, the cartridge was washed with 3 ml water and absorbed substances were eluted with 1.5 ml methanol. The eluent was evaporated to dryness, reconstituted in 150 μl methanol and centrifuged at 13000 rpm for 5 min. The extract was then diluted 10× with methanol:water (1:1 v/v) and 2 μl were injected into LC–MS.

### LC–MS Analyses

The samples were analyzed on an Acquity UPLC system with an LCT premier XE time-of-flight mass spectrometer (Waters, USA) using the LC Column Acquity UPLC BEH C_18_ kept at the temperature of 30°C (50 mm × 2.1 mm I.D., particle size 1.7 μm, Waters, USA) and a two-component mobile phase at the flow rate of 0.4 ml/min. The mass spectrometer was operated in the “W” mode with the capillary voltage set at +2800 (positive ionization mode) or -2500 V (negative ionization mode), cone voltage of +40 or -40 V, desolvation gas temperature of 350°C, ion source block temperature of 120°C, cone gas flow of 50 l/h, desolvation gas flow of 800 l/h, scan time of 0.1 s, and inter-scan delay of 0.01 s. Fragmentation by collision-induced dissociation (CID) was triggered by setting the aperture 1 value at 50 V. Lincomycin quantification was performed using a standard of lincomycin A spiked into the analyte-free cultivation broth at the required concentration. The data were processed using MassLynx V4.1, and the quantification was performed using the QuanLynx application manager (Waters). Tentative identification of the structures was based on the elemental composition determined from accurate mass and isotopic patterns and, for some of the structures, on in-source CID MS fragmentation and comparison of retention times with those of available authentic standards.

### Chromatographic Conditions

(1) For analysis of amino acid precursors: the two component mobile phase, A and B, consisted of 0.1% formic acid and methanol, respectively. The analyses were performed under a linear gradient program (min/%B) 0/5, 5/5, 10/20, 15/50 followed by a 1.5 min column clean-up (99% B) and a 1.5 min equilibration (5% B). The total analysis time was 18 min.

(2) For analysis of lincomycin derivatives: the two component mobile phase, A and B, consisted of 1 mM ammonium formate pH 9.0 and acetonitrile, respectively. The analyses were performed under a linear gradient program (min/%B) 0/5, 1.5/5.0, 10.0/30, followed by a 2 min column clean-up (80% B) and a 2 min equilibration (5% B). The total analysis time was 14 min.

(3) For analysis of reaction mixtures: the reaction mixtures were centrifuged at 13000 rpm for 5 min and analyzed as described above for the analysis of amino acid precursors except for the linear gradient program, which was (min/%B) 0/5.0, 1.5/5.0, 5.5/9.4 followed by a 1 min column clean-up (100% B) and a 1.5 min equilibration (5% B).

### Lincomycin A Quantification

Lincomycin quantification was performed under the same chromatographic conditions as described above for the analysis of lincomycin derivatives. The extract reconstituted in 150 μl methanol was 2× diluted with water and injected into LC. The column effluent was monitored by UV detection at 194 nm. The calibration curve was prepared using a standard of lincomycin A spiked into the analyte-free extract of cultivation broth. The six calibration points in the range 6.25–200 μg/ml were fitted linearly with the determination coefficient of 0.9997. Lincomycin A production was determined for wt and Δ*lmbY* strains in three independent cultures. The method was developed by a modification of a validated method for determination of lincomycin A (Olsovska et al., 2007).

### Purification of Lincomycin Derivatives

Cultivation broth was loaded onto 10 g of Amberlite XAD-4 sorbent (Supelco, USA) pre-conditioned with 100 ml methanol and 100 ml water. The sorbent was washed with 100 ml water and the absorbed compounds were gradually eluted with methanol:water of increasing ratio of methanol (from 20% methanol to 100% methanol, 10% steps, 50 ml fractions). Fractions containing lincomycin derivatives were evaporated to dryness, reconstituted in methanol and injected into the HPLC apparatus equipped with flow controller 600, autosampler 717, and UV detector 2487 operating at 194 nm (Waters, USA). Data were processed with Empower 2 software (Waters, USA). The samples were separated on the Luna C_18_ chromatographic column (250 mm × 15 mm I.D., particle size 5 μm, Phenomenex, USA) with the two component mobile phase, A and B, consisted of 0.1% formic acid and methanol, respectively. The analyses were performed under a linear gradient program (min/%B) 0/5, 31/27.5 followed by a 9 min column clean-up (100% B) and a 9 min equilibration (5% B), at the flow rate of 3 ml/min. The fractions containing lincomycin derivatives were evaporated to dryness, reconstituted in methanol and injected onto the XTerra Prep RP18 column (150 mm × 7.8 mm I.D., particle size 5.0 μm, Waters, USA). The lincomycin derivatives were eluted using the isocratic program with 1 mM ammonium formate (pH 9.0):acetonitrile (83:17 v/v) as mobile phase. The fractions containing the separated lincomycin derivatives were checked for purity (at least 95%, LC with UV detection at 220 nm) and used for NMR analysis.

### NMR Analysis

NMR spectra were recorded on a Bruker Avance III 700 MHz spectrometer (700.13 MHz for ^1^H, 176.05 MHz for ^13^C) in CD_3_OD (99.8 atom% D, VWR Chemicals, Leuven, Belgium) at 20°C. Residual signals of solvent were used as an internal standard (δ_H_ 3.330, δ_C_ 49.30). ^1^H NMR, ^13^C NMR, COSY, HSQC, HMBC, and 1D TOCSY experiments were performed using the manufacturer’s software. ^1^H NMR and ^13^C NMR spectra were zero filled to fourfold data points and multiplied by a window function prior to Fourier transformation. A two-parameter double-exponential Lorentz-Gauss function was applied for ^1^H to improve the resolution, and line broadening (1 Hz) was applied to obtain a better ^13^C signal-to-noise ratio. Chemical shifts are given in δ-scale with digital resolution justifying the reported values to three (δ_H_) or two (δ_C_) decimal places. Coupling constants (*J*) are in Hz. Some chemical shifts were read out from HSQC spectra. They are given to two (δ_H_) or one (δ_C_) decimal places.

### Bioinformatics Tools

Sequences homologous to proteins LmbA, LmbW, LmbX, and LmbY were searched using blastp (http://blast.ncbi.nlm.nih.gov/Blast.cgi; (Altschul et al., 1997)).

## Conclusion

The newly proposed pathway for the biosynthesis of PPL does not have the ambition to bring all desired answers and proofs. We realize that the ultimate confirmations represent a challenging and long-term task due to a limited availability of the relevant substrates as well as the solubility problems with recombinant proteins (LmbA, LmbY). However, the scheme has been proposed with the hope that it can unblock the situation where several decades accepted scheme, clearly contradictory to our current knowledge, struggles to resolve the individual steps of the biosynthesis.

Our scheme is applicable not only for the biosynthesis of PPL (or 2C APD precursor EPL) but also to a large extent for other APD precursors that are biosynthesized through homologous pathways (only a difference in the final reduction step is presumed). Our study thus provides insights into the biosynthetic machinery of three structurally and functionally distinct groups of bioactive compounds: lincosamides, pyrrolobenzodiazepines, and hormaomycin. Because of their bioactivity, all of these compounds are intriguing from the perspective of the pharmaceutical industry. In particular, the antibiotic lincomycin, binding to ribosomes, and the anticancer pyrrolobenzodiazepines, binding to DNA, have been extensively studied from this perspective. Importantly, it has been shown that specific modification of the APD moiety has a significant effect on the bioactivity. Therefore, probing the biosynthetic machinery of these moieties is an important step for the preparation of improved derivatives of these compounds.

## Author Contributions

PJ and RG contributed equally. JJ and ZK designed the experiments. RG proposed the new biosynthetic scheme. PJ, LS, ZK, and SK carried out tests with recombinant proteins. PJ and JN constructed mutant strains. PJ, ZK, RG, LS, SK, LN, and JJ analyzed results. MK carried out NMR analysis. RG, ZK, PJ, LN, SK, and JJ wrote the paper.

## Conflict of Interest Statement

The authors declare that the research was conducted in the absence of any commercial or financial relationships that could be construed as a potential conflict of interest.
