# Supplementary material for: New Concept of the Biosynthesis of 4-Alkyl-L-Proline Precursors of Lincomycin, Hormaomycin, and Pyrrolobenzodiazepines: Could a γ-Glutamyltransferase Cleave the C–C Bond?
Source: Front Microbiol. 2016 Mar 7;7:276. doi: 10.3389/fmicb.2016.00276 (PMC4780272; doi:10.3389/fmicb.2016.00276)
Supplement: Supplementary file 1 [file Data_Sheet_1.PDF]

Supplementary Material

New Concept of the Biosynthesis of 4-Alkyl-L-proline Precursors of Lincomycin, Hormaomycin and Pyrrolobenzodiazepines: Could a γ-Glutamyltransferase Cleave the C-C Bond?

Petra Jiraskova,[‡] Radek Gazak,[‡] Zdenek Kamenik, Lucie Steiningerova, Lucie Najmanova, Stanislav Kadlcik, Jitka Novotna,[†] Marek Kuzma, and Jiri Janata[*]

*** Correspondence:** Corresponding Author, Dr. Jiri Janata, **janata@biomed.cas.cz**

1 Supplementary Figures and Tables

1.1 Supplementary Figures

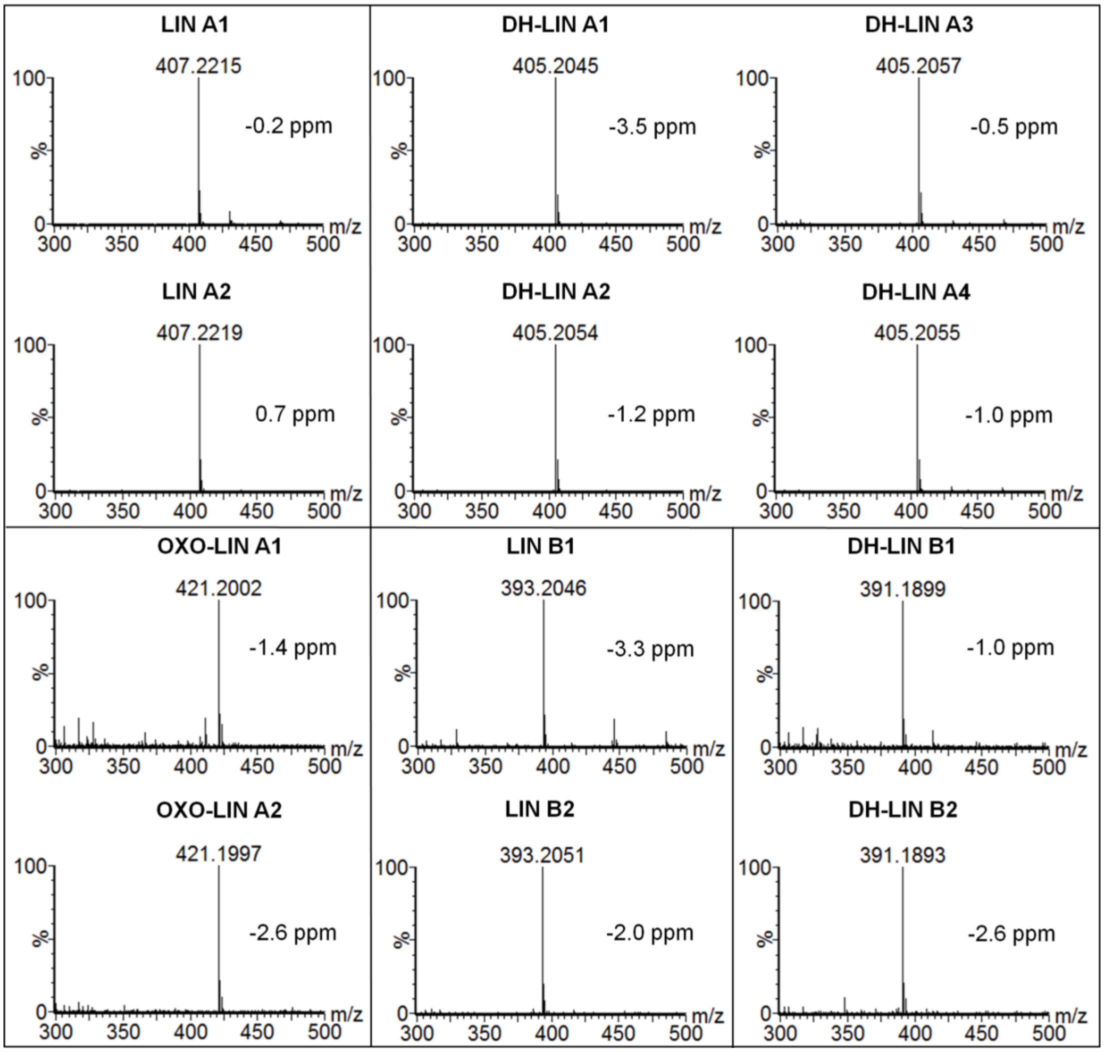

Figure S1A: MS spectra of lincomycin derivatives. Mass error between the measured *m/z* and the theoretical mass of $[M+H]^+$ adduct of the corresponding lincomycin derivative is indicated. Mass error within 5 ppm is typical of the mass spectrometer used. The measured values and the ratio of the isotopic peaks (data not shown) were used to calculate the elemental composition by MassLynx software (Waters)

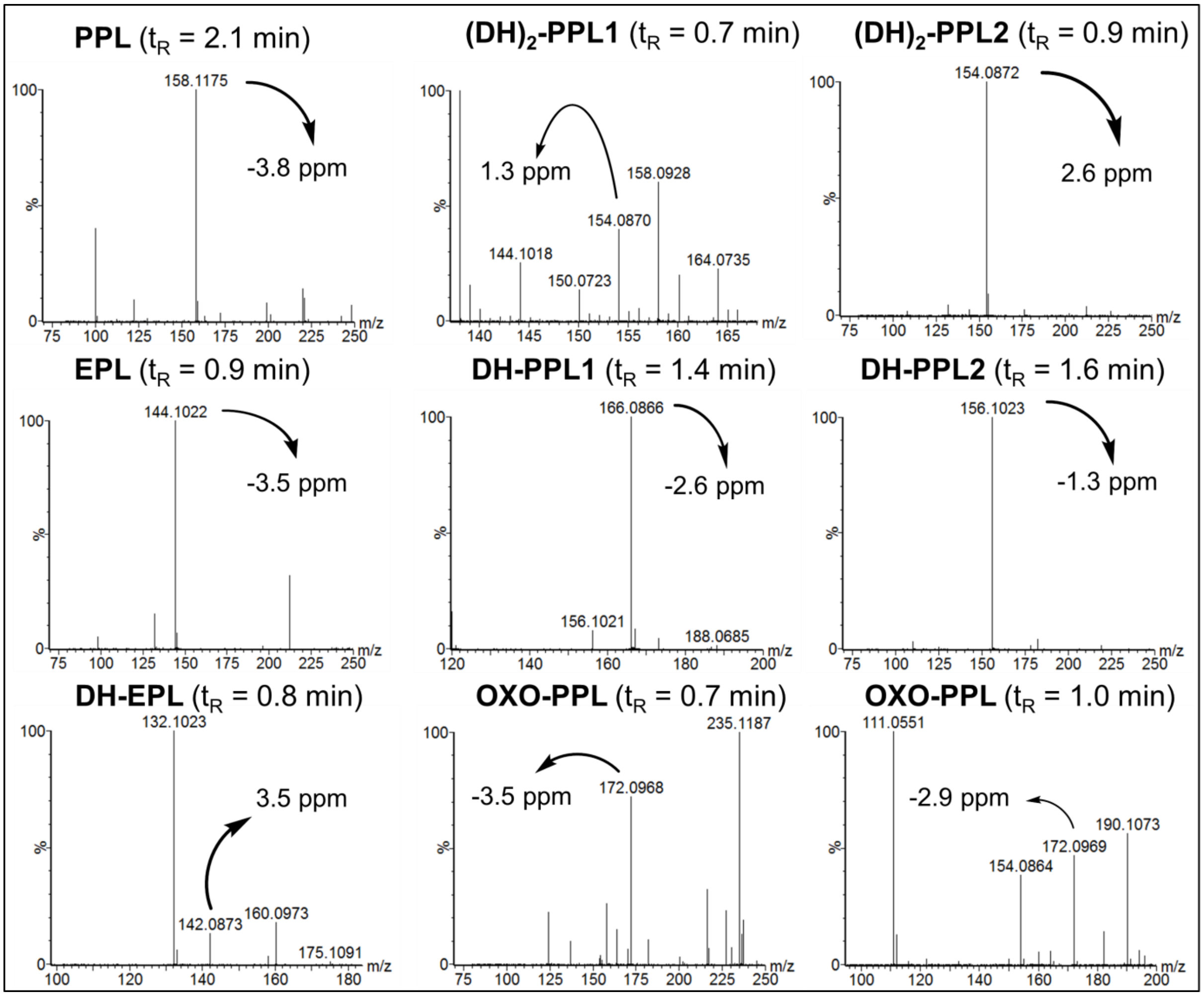

Figure S1B: MS spectra of amino acid precursors. Mass error between the measured *m/z* and the theoretical mass of $[M+H]^+$ adduct of the corresponding lincomycin derivative is indicated. Mass error within 5 ppm is typical of the mass spectrometer used. The measured values and the ratio of the isotopic peaks (data not shown) were used to calculate the elemental composition by MassLynx software (Waters).

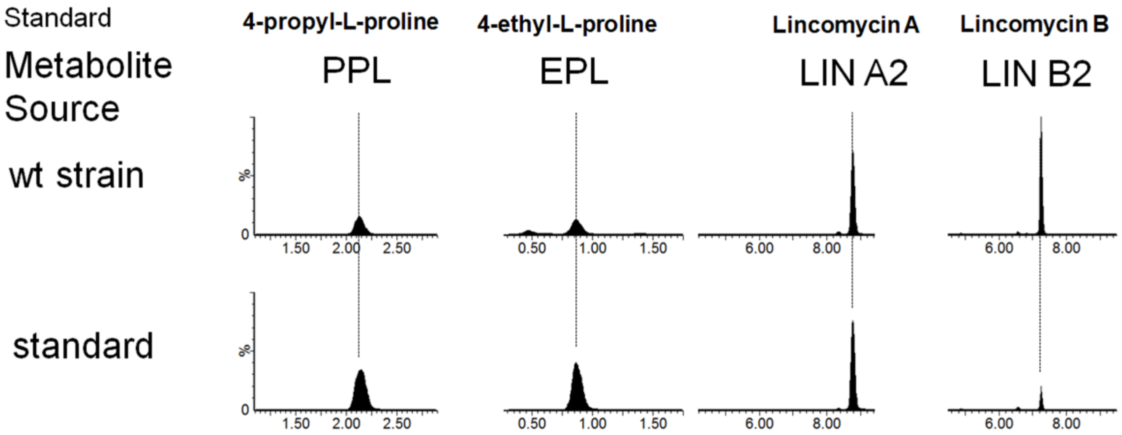

Figure S1C: Comparison of metabolites extracted from culture broth with authentic standards. LC-MS ion extracted chromatograms; Extraction window: 0.025 Da.

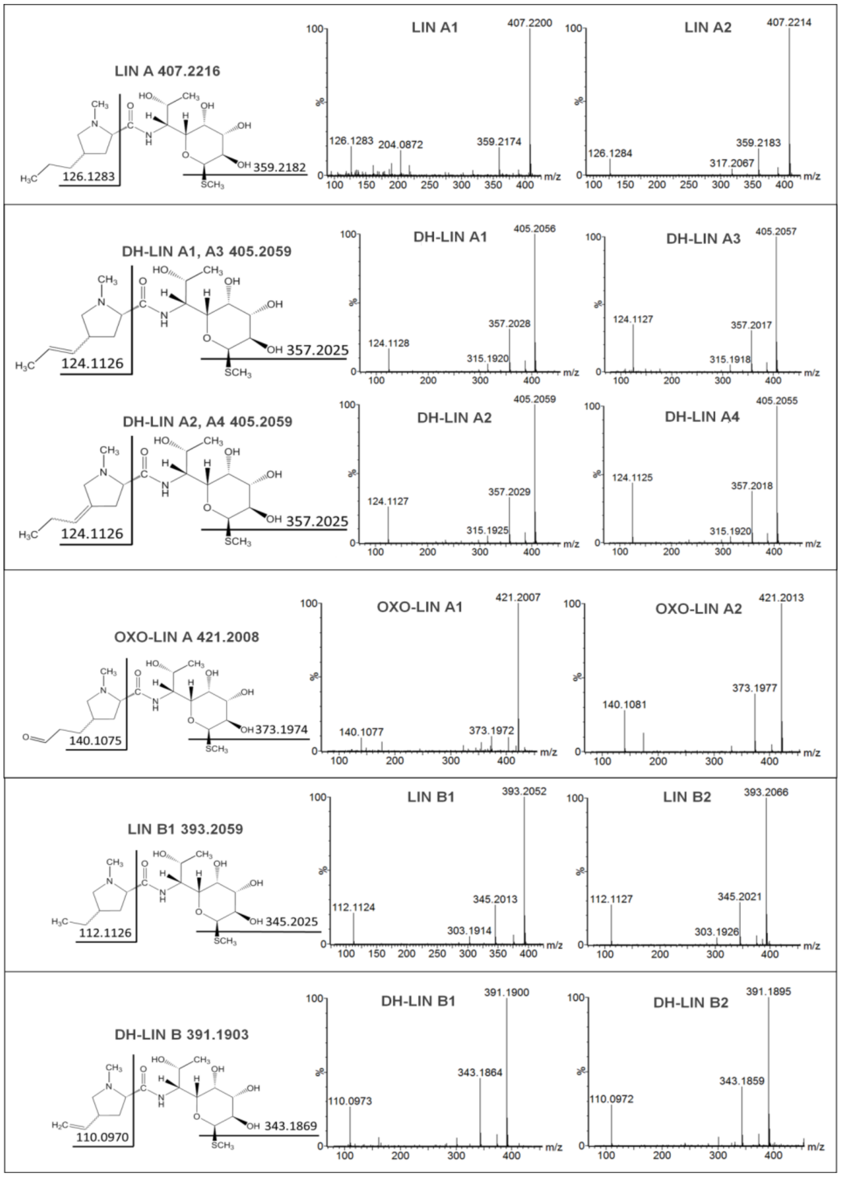

Figure S1D: In-source CID MS/MS spectra of lincomycin derivatives. Theoretical *m/z* of fragments is indicated in the structures. None of the measured *m/z* exceeded the mass error of 5 ppm. The position of the double bonds, the position and character of the oxygen-containing functionality could not be determined from MS experiments. Instead, NMR analysis and/or the individual steps of the biosynthetic machinery described in the main text facilitated the elucidation or tentative elucidation of these features.

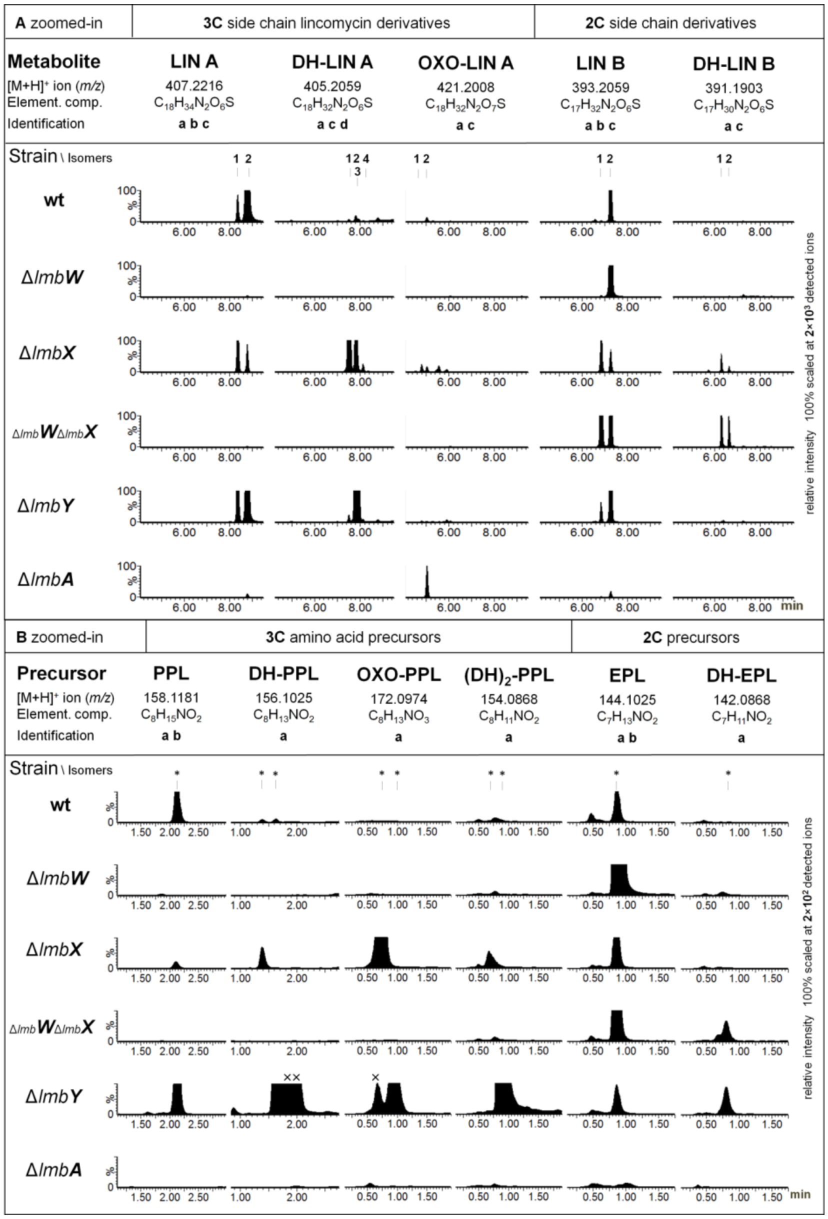

Figure S1E: Zoomed-in production profiles of wt and deletion mutants strains. (A) Lincomycin derivatives (B) Amino acid precursors. LC-MS ion extracted chromatograms; Extraction window: 0.025 Da. Relevant peaks assigned with *; peaks assigned with × do not represent $[M+H]^+$, but a fragment of different species, or their mass error exceeds 5 ppm compared to the theoretical elemental composition. Identification based on: **a** elemental composition calculated from accurate mass and isotopic pattern – **Figures S1A and S1B**, **b** comparison of retention times with authentic standards – **Figure S1C**, **c** in-source CID MS fragmentation – **Figure S1D**, **d** NMR analysis – **Table S1**.

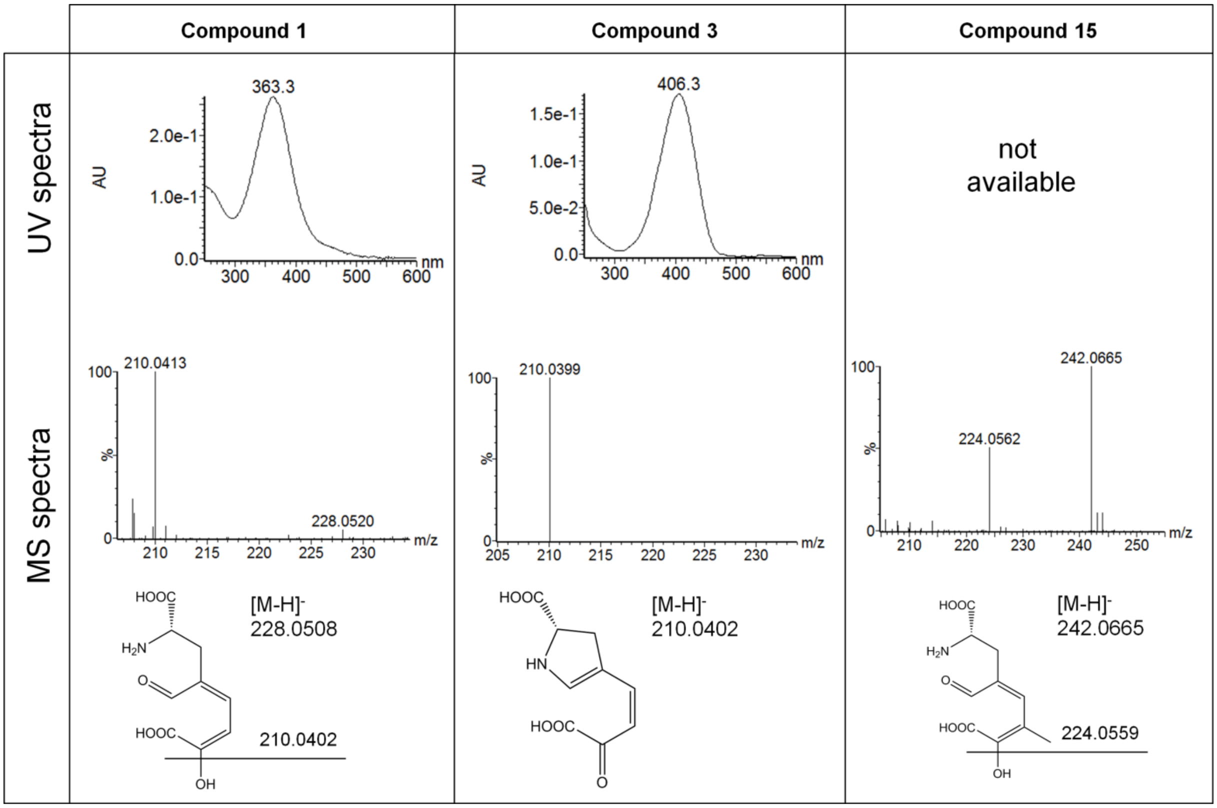

Figure S2: UV and MS spectra of substrate(s) and product of the LmbW assay, corresponding to compounds 1, 3, and 15. UV spectra recorded during LC-MS analysis.

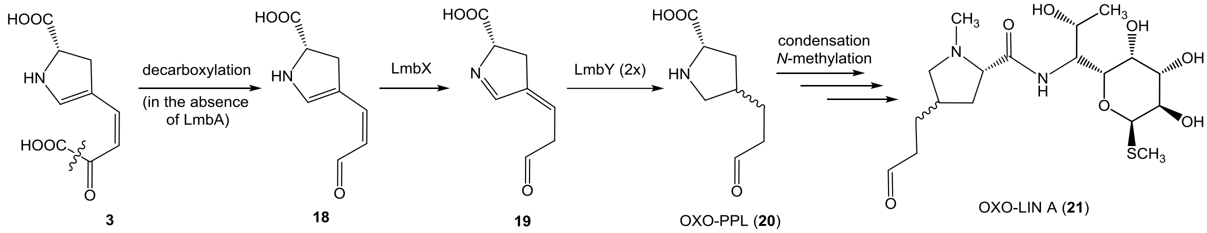

Figure S3: Proposed pathway leading to OXO-LIN A in the absence of LmbA. Compound **21** corresponds to OXO-LIN A according to elemental composition and CID MS fragmentation; however, the structure has not been elucidated by NMR and particularly position and form of the oxygen containing functional group in amino acid moiety is not known.

1.2 Supplementary Tables

Table S1: NMR data.

A. ^{1}H NMR data (700.13 MHz, CD_3OD, 20 °C) of dehydrolincomycins A1-A3

	DH-LIN A1 (**13a**)			DH-LIN A3 (**13b**)			DH-LIN A2 (**14a**)		
position[a]	δ_H	m.	J_{HH} [Hz]	δ_H	m.	J_{HH} [Hz]	δ_H	m.	J_{HH} [Hz]
1	5.255	d	5.6	5.274	d	5.6	5.270	d	5.6
2	4.129	dd	5.6, 10.1	4.144	dd	5.6, 10.1	4.108	dd	5.6, 10.2
3	3.600	dd	3.4, 10.1	3.618	dd	3.4, 10.1	3.581	dd	3.5, 10.2
4	4.021	dd	1.1, 3.4	4.042	dd	1.1, 3.4	3.955	dd	1.2, 3.5
5	4.324	dd	1.1, 7.2	4.366	dd	1.1, 6.8	4.274	ddd	0.4, 1.2, 7.7
6	4.191	dd	7.2, 7.2	4.144	dd	6.8, 7.3	4.241	dd	6.3, 7.7
7	3.994	dq	6.4, 7.2	3.986	dq	6.3, 7.3	4.045	dq	6.3, 6.4
8	1.216	d	6.4	1.222	d	6.3	1.218	d	6.4
1-SCH_3	2.101	s	-	2.107	s	-	2.110	s	-
2’	3.189	br m	-	3.007	dd	4.1, 10.6	3.12[#]	m	-
3’	2.552	ddd	8.2, 8.8, 13.0	2.019	ddd	4.1, 8.3, 12.9	2.872	m	-
	1.686	ddd	5.7, 7.3, 13.0	1.937	ddd	10.2, 10.6, 12.9	2.481	m	-
4’	3.246	br m	-	3.238	br m	-	-	-	-
5’	2.989	br m	-	3.140	ddd	0.6, 6.2, 8.7	3.717	m	-
	2.821	br m	-	2.192	dd	8.7, 10.5	3.10[#]	m	-
6’	5.614	ddq	1.8, 9.6, 10.8	5.276	ddq	1.8, 9.0, 10.8	5.361	m	-
7’	5.471	ddq	0.8, 6.8, 10.8	5.508	ddq	1.1, 6.8, 10.8	2.013	m	-
8’	1.651	dd	1.8, 6.8	1.651	dd	1.8, 6.8	0.994	t	7.5
N-CH_3	2.506	br s	-	2.404	s	-	2.442	br s	-

[a] – for positions numbering see Figure 4 in the main text, [#] – HSQC readout

B. ^{13}C NMR data (176.05 MHz, CD_3OD, 20 °C) of dehydrolincomycins A1-A3

	DH-LIN A1 (**13a**)		DH-LIN A3 (**13b**)		DH-LIN A2 (**14a**)	
position[a]	δ_C	m.	δ_C	m.	δ_C	m.
1	89.96	d	89.7[#]	d	89.9[#]	d
2	69.81	d	69.6[#]	d	69.5[#]	d
3	72.17	d	72.0[#]	d	71.9[#]	d
4	70.94	d	70.7[#]	d	70.6[#]	d
5	70.32	d	70.1[#]	d	70.5[#]	d
6	56.65	d	56.6[#]	d	56.0[#]	d
7	68.38	d	68.2[#]	d	67.8[#]	d
8	19.80	q	19.7[#]	q	18.9[#]	q
1-SCH_3	13.64	q	13.3[#]	q	13.4[#]	q
1’	176.5*	s	178.0*	s	n.d.	-
2’	71.01	d	70.0[#]	d	71.0[#]	d
3’	39.56	t	39.0[#]	t	34.7[#]	t
4’	36.28	d	37.0[#]	d	n.d.	-
5’	63.17	t	63.3[#]	t	62.0[#]	t
6’	135.15	d	132.2[#]	d	124.2[#]	d
7’	125.18	d	126.6[#]	d	23.4	t
8’	13.32	q	13.3[#]	q	14.1[#]	q
N-CH_3	42.15	q	41.7[#]	q	41.4[#]	q

[a] – for positions numbering see Figure 4 in the main text, * – HMBC readout, [#] – HSQC readout

Table S2: List of primers

Primers for gene inactivation		
**Deletion mutant strain**	**Primer**	**Sequence (5' to 3')**
Δ*lmbA*	dAf	TGACCTTCCCGACAACACAGTTATGGTGGTGGACGCATGATTCCGGGGATCCGTCGACC
	dAr	GGCGTCTCTCCGGTGGTTCGCGCGTGTGGCGTGGCGTCATGTAGGCTGGAGCTGCTTC
	cAf	ACCTTCCCGACAACACA
	cAr	GGTCCAGGAAACGGTACA
Δ*lmbW*	dWf	GGATGGCAGGCTTTCACCCTCTCCCAACTGCCCGACATGATTCCGGGGATCCGTCGACC
	dWr	GGCGTGGCGGGCCGGGCCCTTGGGGCCGCCGTCGCGTCATGTAGGCTGGAGCTGCTTC
	cWf	CGCAACTACGGTGAACGA
	cWr	TCAGGGCACATGGAGTCT
Δ*lmbX*	dXf	CGCGCCCATCCTGCACAGCGCACCGGAGGAAGCATGATCATTCCGGGGATCCGTCGACC
	dXr	GAGAAAAGAGCCGCTGACGCAAGGGGCCCTCGGCGACTATGTAGGCTGGAGCTGCTTC
	cXf	CCGGCATCAACGACT
	cXr	CCAGATGGAACGAATTCA
Δ*lmbY*	dYf	GGGCGCGACGAACCATGAGAGGAGAGCGGGCGAACCATGATTCCGGGGATCCGTCGACC
	dYr	GAACACGTTCGGCGCGCCCATCGTGACCCAGGTGTCCGCTGTAGGCTGGAGCTGCTTC
	cYf	GGGCGCCTAACTCTG
	cYr	ACCACCGTCAACTGACTG
	dblaf	CCTGATAAATGCTTCAATAATATTGAAAAAGGAAG
	dblar	AATCAATCTAAAGTATATATGAGTAAACTTGGTCTGACAG
	cblaf	TCTACGGGGTCTGACGCTCA
	cblar	TCTTAGACGTCAGGTGGCAC
Δ*lmbW*Δ*lmbX*	dWhygf	TGCGGCAAACAAGCCCTTCAGACGGACCGAGGTGCCATGATTCCCCTGCTCGCGCA
	dWhygr	CGCGGCACCAGGTCGTAGAAGCACATCCGGGGGTCAGGCGCCGGGGGCGGTGT

Primers for complementation of *S. lincolnensis* Δ*lmbA*	
**Primer**	**Sequence (5' to 3')**
fA10257N	ATACATATGCACCACCACCACCACCACCTTCCATCGAAGCCGGAGC
rA10257N	AGTCTCGAGTCATCCGCAGATCGCGTAGG
sequencing primer	AGACCTACCGGCAGCTCCT

Primers for heterologous expression of selected genes		
**Gene**	**Primer**	**Sequence (5' to 3')**
*lmbX**	ExXf	CATATGATCGTGGTCCCGTTC
	ExXr	AACCTCGAGCTACACGGAAGCGACCG
lmbW	ExWr ExWf	GCATATGACAGCCGTTCGGCAAAG<br>ATTCTCGAGCCGCCGCGGCACCAGGT

Primers for gene inactivation („d“ = deletion primer, „c“ = checking primer); scar sequence in Δ*lmbA*, Δ*lmbX*, Δ*lmbW* was introduced by protoplast transformation, scar sequence in Δ*lmbY* was introduced via conjugation requiring replacement of cosmid *bla* gene with pIJ798 cassette containing *oriT* and hygromycin resistance. For preparation Δ*lmbW*Δ*lmbX* primer pairs dWhygf/r, dXf/r, cWf/r,

cXf/r were used. * Primers were used both for heterologous expression of *lmbX* and complementation experiment.
